# Color disparities in cognitive aging among Puerto Ricans on the archipelago

**DOI:** 10.1016/j.ssmph.2021.100998

**Published:** 2021-12-13

**Authors:** Mao-Mei Liu, Michael Crowe, Edward E. Telles, Ivonne Z. Jiménez-Velázquez, William H. Dow

**Affiliations:** a Department of Demography, University of California Berkeley, 2232 Piedmont Avenue, Berkeley, CA, 94720, USA; b Department of Psychology, University of Alabama, Birmingham, Campbell Hall, Rm 334, 1300 University Blvd., Birmingham, AL, 35233, USA; c Department of Sociology, University of California Irvine, 4171 Social Science Plaza A, Irvine, CA, 92697-5100, USA; d School of Medicine & Department of Medicine, University of Puerto Rico, Medical Sciences Campus PO Box 365067, San Juan, PR, 00936-5067, USA; e School of Public Health, University of California Berkeley, 2121 Berkeley Way #5324, Berkeley, CA, 94720, USA

**Keywords:** Dementia, Racial disparities, Race, Color, Puerto Rico, Cognitive health

## Abstract

This research seeks to contribute new understanding of color disparities and gender in cognitive aging among older adults residing in Puerto Rico. We use the island-representative Puerto Rican Elderly Health Conditions (PREHCO) longitudinal study that measures cognitive health at baseline and cognitive decline between waves. In pooled models, we discern little or no color disparities in cognition at baseline. Sex-stratified models of baseline cognition indicate that Trigueño men slightly outperform white men. In contrast, color disparities in cognitive decline are apparent. In just four years between the two waves of PREHCO, on a 20-point cognitive test scale, Black men experienced 0.78 more points of cognitive decline, while Trigueño men experienced 0.44 more points of cognitive decline than white men in Puerto Rico. Mestiza women experience 0.80 less points of cognitive decline relative to white women. Nearly all of the color/race association with cognitive decline appears to be independent from health behaviors and conditions, individual human capital attainment, and family background. While lower-status color groups more frequently report discrimination, discrimination does not mediate the impact of color/skin tone and cognitive performance, suggesting the importance of further research on the role of broader dimensions of life course structural racism.

## 1 Background

**Fig. 1:**
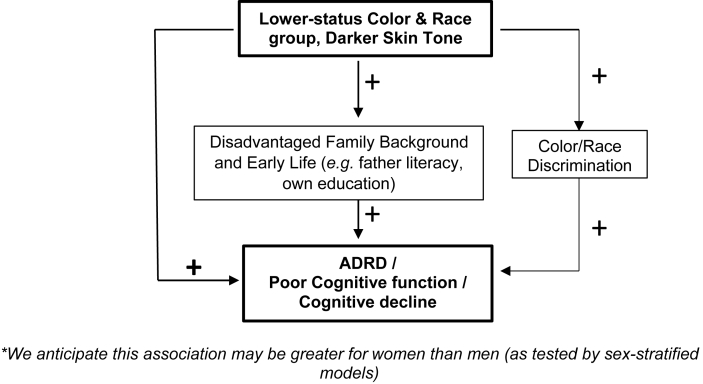
Conceptual Framework*. *We anticipate this association may be greater for women than men (as tested by sex-stratified models).

Racial disparities in Alzheimer's disease and related dementias (ADRD) and cognitive aging are stark in the U.S. and related to life course processes and social contexts (e.g. Glymour & Manly, 2008). As legacy of conquest and slavery in the United States and the Americas, institutionalized racial inequalities in health, education, residence, mass incarceration, employment, and wealth endure, indeed thrive, in our societies (Telles, 2014; Williams & Collins, 2016). In the U.S., Black and Latino individuals experience higher ADRD risk and prevalence than White individuals (Association, 2019; Matthews et al., 2019; Steenland et al., 2016), but the reasons are not yet well understood (Mehta et al., 2008). Scholars of racial health disparities most often focus on Black and White differentials in ADRD (Steenland et al., 2016; Weuve et al., 2018; Yaffe et al., 2013) and pay less attention to Latino populations and intragroup heterogeneity. Studies of ADRD among Latinx or Hispanic individuals (Clark et al., 2005; Vega et al., 2017) usually do not examine the possible roles of race, color and origin. This paper examines color/racial disparities in cognitive aging, and the life course determinants of those disparities (see Fig. 1 Conceptual Framework).

We examine the racial stratification of cognitive health for a specific, phenotypically diverse Latino population: Puerto Ricans who live in Puerto Rico. Prior research focused on African Americans has found that skin tone is associated with intragroup disparities in education, labor market and health (Keith & Herring, 1991; Monk Jr, 2015). The potential role of color and skin tone for Latinos has been less explored. Existing evidence of Puerto Ricans is limited but suggests gradients of health by race and color (Borrell, Crespo, & Garcia-Palmieri, 2007; Gravlee et al., 2005). We seek to contribute new research on whether and how color and race are associated with cognitive health for older Puerto Ricans using island-representative data. We also seek to understand whether and how these processes may be affected by gender and reflect life course pathways of family and individual advantage.

Studying race and health in Puerto Rico is important for several reasons. First, Puerto Rico is a territory of the United States and the four centuries-long history of Spanish colonialism and hundreds of years of slavery continue to influence population health and well-being. Historical disinvestment related to its colonial statuses deeply impacts Puerto Ricans’ health and health systems (Benach et al., 2019). Second, Puerto Rico is part of Latin America. Like many other Latin American nations and in contrast to the U.S., Puerto Rico has significant race mixture and national narratives of mixture (Duany, 2003) and racial harmony. Also, as in other countries in Latin America, race is more fluid and numerous intermediate terms are in use in Puerto Rico (Duany, 2003, pp. 238). Finally, the population of Puerto Rico is rapidly aging, but our knowledge of the health status and cognitive health of Puerto Rican older adults living in the archipelago remains limited (Pérez & Ailshire, 2017).

### 1.1 Race, color and cognitive health

Race is a complex, multi-dimensional construct and can be measured in several ways. The dimensions of race require different social science tools: racial identity (open-ended question); racial self-classification (closed-ended survey question, e.g., Census categories), observed or ascribed race (usually interviewer-rated); reflected race (self-reports of the race you believe others assume you to be); phenotype (usually interviewer observations of skin tone or other features); and racial ancestry (self-reports or genetic testing results) (Roth, 2016). Racial health disparities research usually relies on racial self-classification. The U.S. Census two-question format is widely employed and elicits Latino/Hispanic identity and racial self-classification separately. As the latter does not include a Latino category, Latinos can self-identify racially as black, white, or Asian, Native American and ‘other’ but not Latino. These racial categories fall short for U.S. Latino populations (Rodriguez, 2000) whose diversity does not easily fit standard schema (Borrell, 2005) and who often understand (and identify with) race as Hispanic or Latino, even though they phenotypically vary from being perceived as white, black or mixed (Telles, 2018). Indeed, observed or ascribed race and phenotype are considered to be essential for examining discrimination and racial disparities (Landale & Oropesa, 2005), but are rarely measured in population-based health surveys.

Existing research on race as phenotype and health focuses on African Americans and their skin tones. Among African Americans, having darker skin tone is associated with lower educational attainment (Hersch, 2006; Loury, 2009), worse labor market outcomes (Goldsmith et al., 2006; Keith & Herring, 1991) and sometimes poorer health. Some studies find darker skin tone to be associated with hypertension (Harburg et al., 1978; Monk Jr, 2015), while a few studies find negative associations with mental health (Louie, 2020; Monk Jr, 2015), but not overall health (Borrell et al., 2006; Monk Jr, 2015). Discrimination appears to be a primary mediator between skin color and health (Monk Jr, 2015; Perreira & Telles, 2014).

Studies examining skin tone and health within Latino/Hispanic populations are more limited but interest is growing (Cuevas et al., 2016). Previous research finds skin tone disparities in education among Mexican Americans (Murguia & Telles, 1996) and occupational status among Mexican Americans and Cuban Americans, but not Puerto Ricans (Espino & Franz, 2002). In other population-based studies, darker skin tone appears to be associated with poorer self-reported health in several Latin American countries (Perreira & Telles, 2014) and poorer mental health already among Latino *first graders* in the U.S. (Calzada et al., 2019).

Studies of phenotype and cognitive aging are extremely limited. Indeed, we do not know yet of any study that examines how race or color is associated with neurocognitive functioning among Latinos in the mainland U.S. or Puerto Rico, or among African Americans in the U.S. Instead, we are motivated by associated research on the cognitive aging of white- and Black-identifying Latinos. First, Black Latinos in the U.S. are more likely than white Latinos to report ADRD-related health conditions like diabetes (Borrell, Crawford, & Dallo, 2007) and hypertension (Borrell, 2006). Second, there is evidence that risk of ADRD and other health conditions may be higher in those with chronic stress, possibly reflected by Black and white Latinos differences in mental health experiences. Black Latinos report higher levels of psychological distress than White Latinos (Mena et al., 2019). Research to-date of how color/race are associated with neurocognitive functioning is indeed limited.

### 1.2 Color, health and gender in Puerto Rico

Puerto Ricans use a multitude of color categories (Duany, 2003; Godreau, 2000), but previous influential work (Gravlee, 2005) closely examined the core emic categories of color in Puerto Rico. The color/race categories we employ in this paper - *Negro* (black), *Blanco* (white), *Trigueño, Mestizo* – greatly overlap with the shared understanding or social classification of color that Gravlee found in his influential study in southeastern Puerto Rico (although his classification included Jabao and not Mestizo). Such understanding of color/race relied primarily on contrasts in skin color and hair texture. While Afro-descendant populations are more concentrated in certain geographic areas of Puerto Rico, there is significant skin tone diversity throughout the island. Puerto Rico's skin tone diversity contrasts with the dominant national narrative – as evident in school curriculum and elsewhere – that elevates the idea of the Puerto Rican people as a mixed Taino (indigenous) and European people (Godreau et al., 2008). Blanco (white) is an esteemed category whose boundaries expanded in early 20^th^ century Puerto Rico (Loveman & Muniz, 2007). Negro (Black) is a maligned category that is a recent focus of organizing and affirmation (Lloréns, 2018). *Trigueño* (literally wheat-colored) is an intermediate (between black and white), fluid term which is often considered equivalent to mulatto, but can also be used as a euphemism for Black (Vargas-Ramos, 2005). These color categories contrast with the US Office of Management and Budget's (OMB) racial categories that have historically lacked mixed options (Godreau et al., 2010). In addition, while skin color had indeed been strongly associated with socioeconomic position in several other Latin America countries (Perreira & Telles, 2014; Telles et al., 2015; Villarreal, 2010), our study is among the first to use island-representative data to assess this in Puerto Rico (Caraballo-Cueto & Godreau, 2021).

Despite new attention to health disparities in Puerto Rico in the wake of Hurricane Maria and recent earthquakes (Kishore et al., 2018; Ma & Smith, 2020) and renewed focus on historical disinvestment and systemic failures of Puerto Rico's health care system related to its colonial status (Benach et al., 2019), health disparities research focusing on race and skin tone in Puerto Rico is quite limited to date. Existing evidence suggests that the influence of interviewer- or self-rated racial identities on health depends on socio-economic standing, residence, and gender. Gravlee and colleagues' influential studies of a community-based sample in southeastern Puerto Rico (Gravlee et al., 2005, 2009) found that interviewer-rated color/race classification was associated with blood pressure outcomes. *Negros* with average or above average socio-economic status had higher blood pressure relative to *Blancos*, but differences were not found among individuals of low socio-economic status (Gravlee et al., 2005). *Trigueños* and *Blancos* had similar blood pressure levels. In contrast, they found no association between machine-rated skin pigmentation and blood pressure. In a longitudinal study of Puerto Rican men in northeast Puerto Rico, Borrell and colleagues found that interviewer-rated darker skin tone (using palettes) was associated with an increased risk of all-cause mortality among men in urban areas, but no associations were found for rural residents nor for CVD-related mortality (Borrell, Crespo, & Garcia-Palmieri, 2007). At least one study includes women: while the interviewer-rated skin tones of island-dwelling (and New York City-dwelling) Puerto Rican mothers were *not* associated with low infant birthweight (Landale & Oropesa, 2005), mothers' skin tone was associated with low infant birthweight for mothers living in other Eastern states. In a recent analysis of the archipelago-representative 2016 Puerto Rico Behavioral Risk Factor Surveillance System (PR-BRFSS), researchers found evidence that the most light-toned Puerto Ricans report better overall health than the most dark-toned Puerto Ricans (Caraballo-Cueto & Godreau, 2021). To our knowledge, no archipelago-wide population-level study exists of race and color disparities in Puerto Ricans' cognitive health. Needed are studies that assess the salience of color/race disparities in health for men *and* women; rely on Puerto Rican-wide population data; and consider a broader set of health outcomes, including cognitive aging. Our study intends to help remedy this gap. Overall, we expect that *historically marginalized groups (e.g. Black, Trigueño) will have poorer neurocognitive functioning (& experience greater cognitive decline over time) than the more privileged class (white) in Puerto Rico.*

In Puerto Rico, health outcomes differ for men and women, and gendered health processes may be related to race and skin tone. Puerto Rican women have poorer overall health than men living on the island and are more likely to have heart disease, hypertension, diabetes and overall poor self-rated health (Pérez & Ailshire, 2017). How might race and gender disparities intersect? Will men and women experience race- and color-based cognitive health disparities similarly? We explore these potential gender-based differences through sex-stratified regression models throughout our paper (while less than ideal, sex is the only measure related to respondent gender in PREHCO). As prior research on gendered experiences of skin tone in Puerto Rico (López, 2008) and of Puerto Ricans in the mainland U.S. is very limited (Quiros & Dawson, 2013), we draw on other gender and skin tone research. Prior research of African Americans finds that women and men's experiences of skin tone stratification are different and help to reinforce traditional gender norms (Thompson & Keith, 2001): for example, skin tone especially impacts women through attractiveness ratings (Hill, 2002) and status attainment (Ryabov, 2019) to the detriment of darker-toned women. Indeed, recent studies find that color differences may especially impact African American women's health (Hargrove, 2019) and women's marriage prospects (Hunter, 2007; Monk Jr, 2014). Both African Americans in the U.S. and older adults in Puerto Rico grew up in and navigate social and economic contexts that are influenced by traditional gendered norms and colorism (Dixon & Telles, 2017; Quiros & Dawson, 2013). As such, we expect that *older Puerto Rican women – like African American women – would experience stronger impacts of race and color than Puerto Rican men. Alternatively, it is possible that race/skin tone and colorism are simply not as relevant in the Puerto Rican context, and men and women would experience it similarly.*

### 1.3 Explaining color disparities in cognitive health

We anticipate that color/race disparities in cognitive health among Puerto Rican older adults are likely linked to family background and human capital exposures that operate over the lifespan. First, low education is a major risk factor for ADRD (S. Norton et al., 2014) and is one pathway through which structural racism and colorism are expressed in societies. Skin tone is a significant predictor of educational attainment for African Americans in the 20^th^ and 21^st^ century (Keith & Herring, 1991; Monk Jr, 2015). Indeed, inequality by skin tone within Black communities appears comparable in size to inequality between the Black and white populations in general (Monk Jr, 2015). Educational attainment for Latinos in the United States and beyond also appear to be stratified by skin tone. The lightest-toned Mexican Americans have about 1.5 more years of education than the darkest-toned Mexican Americans in data gathered in 1979 (Murguia & Telles, 1996). Studies of nationally representative data in Latin America indicate that lighter-toned individuals are more highly educated than their darker-toned counterparts (Perreira & Telles, 2014; Telles et al., 2015). Echoes of such results have been found for men in northeast Puerto Rico (Borrell, Crespo, & Garcia-Palmieri, 2007) and Latino adolescents in the U.S. (Thompson & McDonald, 2016).

Prior research offers evidence that the impact of color on health is at least partially mediated through important socioeconomic categories like education or income. A recent nationally representative U.S. study of mortality finds significant skin tone disparities among higher educated African Americans but not for the less-educated (Stewart et al., 2020). These mortality findings are preceded by studies of income and blood pressure. While income and blood pressure are inversely related among light-toned African Americans, no significant gradient is discerned among dark-toned individuals (Sweet et al., 2007). In southeastern Puerto Rico, high blood pressure appears to be stratified by skin tone for high-status men, but not among lower-status men (Gravlee et al., 2005). We anticipate that family background and human capital advantages will partially explain color/race disparities in cognitive health and seek to test three hypotheses: *c1. Individuals of historically marginalized groups will have less education and less literate fathers; c2. Individuals whose fathers were literate will experience cognitive health protections over and beyond their own educational achievements; c3. Higher educated individuals will experience cognitive health advantages.*

Color stratification of health may also operate through experiences of discrimination. Previous research establishes that individuals who live and work in white-dominant spaces are more likely to experience discrimination and related stressors (Hunter, 2007; Jackson et al., 1995; Jackson & Stewart, 2003). Perceived discrimination has been associated with a range of poor mental and physical health outcomes (for review, see Williams & Mohammed, 2009). Darker skin tone is strongly associated with more experiences of discrimination for African Americans (Monk Jr, 2015) and Latin Americans (Perreira & Telles, 2014). However, while measures of perceived discrimination predict important health outcomes for African Americans (Monk Jr, 2015), they appear to explain little of the association between skin color and self-rated health in Latin America (Perreira & Telles, 2014). To the best of our knowledge, scholars have yet to examine how color and perceived discrimination are associated with health for representative samples of Latino communities in the U.S or Puerto Rico. Given previous research findings, *we hypothesize that discrimination measures partially explain color/race disparities in cognitive health.*

## 2 Current study

In this paper, we seek to describe racial and migrant health disparities in cognitive aging using island-wide Puerto Rican Elderly Health Conditions (PREHCO) study and test hypothesized mediators of race/skin tone and ADRD among Puerto Ricans (see Fig. 1).

a) Historically marginalized groups (e.g. Black, Trigueño) will report poorer neurocognitive functioning and greater cognitive decline than the privileged class (white).
b) For gender and race/color, we have two competing hypotheses: b1. Women experience stronger race and color-based differences in cognitive health b2. Women and men experience comparable race and color-based differences in cognitive health.
c) Family background and human capital advantages will partially explain the impact of color/race on cognitive health: c1. Individuals of historically marginalized groups will have less education and less literate fathers c2. Individuals whose fathers were literate will experience cognitive health protections over and beyond their own educational achievements c3. Higher educated individuals will experience cognitive health advantages.
d) We hypothesize that discrimination will partially explain the impact of color/skin tone and cognitive health: d1. Individuals of historically marginalized color/race groups will experience more color discrimination d2. Individuals who experience color discrimination will have poorer cognitive health.

## 3 Data and methods

### 3.1 Data

We use data from the Puerto Rican Elderly Health Conditions (PREHCO) project (https://www.icpsr.umich.edu/web/DSDR/studies/34596). PREHCO is an archipelago-representative, longitudinal panel of community-dwelling Puerto Rican older adults 60 years and older. We use data from both the first (2002–2003) and second waves (2006–2007). PREHCO employed a multistage, stratified sampling strategy that oversampled regions heavily populated by Afro-descendant individuals, and individuals older than 80. The PREHCO questionnaire included modules on demographic characteristics, health status and conditions, cognitive performance, labor and economic status, and family structure, among others. All interviews were conducted in Spanish. For additional information on the study and its design, please see (McEniry, 2011; McEniry & Palloni, 2010; Palloni et al., 2005, 2020).

4291 respondents participated in PREHCO's 2002–2003 baseline survey. Given the design of the study, we focus on individuals who completed the baseline questionnaire without a proxy and who self-reported color/race (n = 3634), as proxies did not report respondent color/race or experience of discrimination. Appendix 1 displays sample statistics by proxy status. Proxy respondents are much older than non-proxy respondents (77.3 vs 69.9 years old) and are less educated, less likely to be married, and do less exercise. Next, we excluded 1.6% (n = 57) respondents who are missing at least one of the baseline covariates. Our analytical sample for baseline cognition is 3575 respondents. In the analyses of cognitive decline, we exclude 894 respondents who died or were otherwise lost to follow-up and/or missing cognitive assessments for wave 2, resulting in a total analytical sample for cognitive decline of 2681.

### 3.2 Measures

#### 3.2.1 Cognitive health

**Fig. 2:**
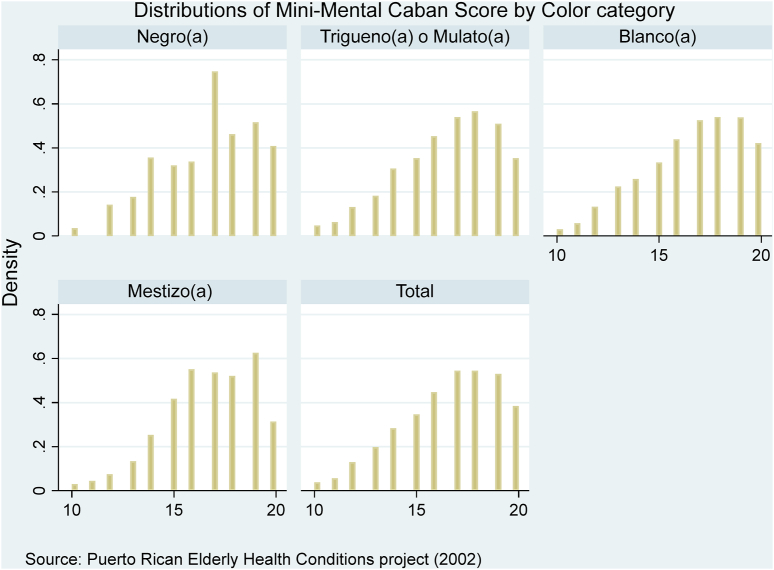
Distributions of mini-mental Cabán scores by color.

PREHCO uses the Mini-Mental Cabán or MMC, which is a validated instrument for measuring cognition for older Spanish speakers (Sánchez-Ayéndez et al., 2003). The MMC includes orientation, verbal memory (immediate and delayed recall of word list), visual memory, visuospatial/executive function (clock drawing, copy pentagons), abstraction and comprehension. The MMC score is a continuous measure and has a maximum of 20 points. We employ the baseline MMC score as our measure for cognition at baseline. Individuals who scored below a pre-determined cutoff (<11) at baseline were then interviewed using a proxy and did not answer questions about discrimination and self-identified color/race. As such, the lowest scoring individuals were excluded from our analysis of baseline cognition. Fig. 2 shows the MMC score distribution overall and by color categories. All distributions are skewed towards the left. The long left tails do not appear significantly truncated by the MMC cut-off. Cognitive decline is the 2002 baseline MMC score subtracted by the 2006 MMC score. We can include even the lowest scoring individuals in 2006 in the analysis of cognitive decline since respondents reported color/race at baseline in 2002. No cutoff was applied to the 2006 MMC scores for the purposes of current analyses.

#### 3.2.2 Color

In this paper, we used PREHCO's color/race categories: *Black*, *Trigueño* (which also includes *Mulatto/a* and Mixed), *white*, *mestizo/a*, and excluded the small ‘other’ category. With the preface “I am going to ask you a series of questions about skin color. In Puerto Rico we have a lot of mixture and we would like to know”, PREHCO elicits respondent-rated race and color with the interviewer asking respondents: “If you had to classify and describe yourself using one of the following categories, which would you choose? Black, mulatto/a, mixed or wheat-colored *(trigueño/a)*, white, mestizo/a, or using some other category?” The survey instrument explicitly notes “‘*Trigueño*’ refers to the mix of black and white; ‘Mestizo’ means the mix of Indian and white.” Previous research in southeast Puerto Rico (Gravlee et al., 2005) which employed similar terms found that social classification of color/race – but not machine-rated skin tone – was associated with blood pressure. Previous unweighted analyses of PREHCO by Crowe et al. (Crowe et al., 2016) show that Trigueño-identifying individuals have lower cognitive function and faster cognitive decline than white individuals.

#### 3.2.3 Discrimination

Immediately after the color/race question, PREHCO interviewers asked respondents “Have you ever felt discriminated against because of your skin color? Would you say this has occurred frequently, sometimes, or never?” In our analysis, we distinguish between ever experiencing color discrimination (frequently or sometimes) vs never experiencing discrimination.

#### 3.2.4 Covariates

We employ a multifaceted predictive model of cognitive health that includes factors previously associated with cognitive decline: key demographics, human capital, family background, health conditions, health behaviors (Kalaria et al., 2008; Livingston et al., 2020). In all models, we control for foreign-born status (whether born outside Puerto Rico), age, age squared, sex, marital status, and whether the respondent grew up in the countryside before age 18. We include age squared to account for ADRD non-linearities in age (Gao et al., 1998). Measures of education include respondent's own level of education (6^th^ grade or less; 7^th^ to 12^th^ grade; more than 12^th^ grade) and whether the respondent's father could read. Chronic health conditions include diabetes (respondents reported whether a doctor had ever told them they had diabetes) and hypertension (respondents reported whether a doctor had ever told them they had hypertension). Health behaviors include whether the respondent had ever smoked 100 cigarettes and whether the respondent exercised three of more times a week in the previous year.

### 3.3 Methods

Our primary aim is to compare the cognitive health and experiences of Black-, Trigueño- and Mestizo-identifying Puerto Ricans to white-identifying Puerto Ricans. First, we explored the sample characteristics by group. Second, to examine color disparities in experiences of discrimination, we estimated logistic regression models to predict perceived color discrimination. Third, we estimated generalized linear models to predict cognitive test performance at baseline (2002), as well as cognitive decline between 2002 and 2006. These generalized linear models are flexible generalization of OLS which allow for response variables having error distributions that are not normal distributions. Fourth, we employ Baron and Kenny's approach to mediation (Baron & Kenny, 1986): we include our proposed mediators (e.g. education, discrimination) in a model and assess whether inclusion of these variables results in an appreciable reduction in magnitude of the parameter estimate. Previous scholars have advocated for such approaches to study racial disparities (VanderWeele & Robinson, 2014; Ward et al., 2019). To minimize misinterpretation of covariate coefficients (Westreich & Greenland, 2013), we have hidden them in the main tables. Full tables are included in the Appendix. We account for the complex survey design of PREHCO by employing sample weights (wave 1 wt - FAC_T - for cognition and covariates measured at baseline; and longitudinal wave 2 wt - FACTORTC - for cognitive decline) and Stata's *svy* commands as we seek to make inferences that are generalizable to the Puerto Rican population. Robust standard errors are reported throughout. We used Stata version 15 to conduct analyses.

## 4 Results

### 4.1 Sample characteristics and race-based disparities

The study sample (n = 3575) reflects the educational and racial diversity of Puerto Rican older adults (see Table 1). Nearly all individuals identify either as Trigueño/a & Mulato/a (hereafter Trigueño) (42.0%) or White (45.5%), with smaller proportions identifying as Black (5.5%) or Mestizo (6.6%). Of every five older adults, two have a primary education or less; two have at least some junior high school education; and one has beyond high school education. In terms of cognitive health, results are mixed. While there are no significant differences in baseline neurocognitive scores by race/color, change in cognitive health between survey waves is different according to race/color. Black and Trigueño individuals experience larger declines in cognitive scores between waves of the PREHCO survey. Overall group differences in smoking and exercise are statistically significant while self-reported health, hypertension and diabetes differences are not. While Black Puerto Ricans report healthier behavior (they are least likely to report ever smoking (26%) and among the most likely to report exercising three times a week (46%)), Black Puerto Ricans are also most likely to report adverse health conditions associated with poor cognitive health: hypertension and diabetes.

**Table 1:** Sample Statistics of Puerto Rican Older Adults by Color/Race presented as Weighted Percentages (%) and Means (Standard Errors), PREHCO (2002, 2005).

	Full sample	Negro	Trigueño mulato	Blanco	Mestizo	
**Cognitive health outcomes**						
MMC score	16.9 (0.1)	16.4 (0.3)	16.9 (0.1)	16.9 (0.l)	17.0 (0.2)	
Cognitive decline (wave1 – wave2)	0.8 (0.1)	−1.4 (0.3)	−1.0 (0.1)	−0.8 (0.1)	−0.3 (0.3)	*
**Health behaviors**						
Hypertension (self-report) (%)	58	66	60	56	56	
Diabetes (self-report) (%)	28	39	28	28	24	
Ever smoke (%)	34	28	33	33	39	
Exercise (%)	43	45	42	43	48	
**Education** (%)						***
6^th^ grade or less	40	37	45	36	38	
7^th^ to 12^th^ grade	40	43	42	38	37	
More than 12^th^ grade	20	20	13	26	25	
**Discrimination (skin color)**						
Ever experienced	5	14	6	2	10	***
**Individual factors**						
Age	69.1 (0.2)	71.2 (1.4)	68.7 (0.3)	69.2 (0.2)	69.2 (0.6)	
Female (%)	56	55	60	56	47	***
Foreign-born (%)	4	3	2	5	7	**
Married (%)	53	40	52	55	61	
**Family background**						
Father literate (%)	66	65	60	70	67	*
Father literacy unknown	6	6	7	5	7	
Lived in Countryside before age 18 (%)	54	48	59	51	49	*
**Number of observations (unweighted)**	3575	197	1499	1627	235	
% of study sample		5.5%	41.9%	45.5%	6.6%	

Source: Puerto Rican Elderly Health Conditions project (PREHCO).

Notes: p value for differences among color/race groups. *p < 0.05, **p < 0.01, ***p < 0.001.

Puerto Rican older adults experience color/race disparities in terms of family background, opportunities, and experiences of discrimination. In Puerto Rico, Trigueño older adults grew up in less resourced families and settings. About three out of every five Trigueño older adults grew up in the largely impoverished countryside during their childhood, while only half of other groups did. In terms of family background, 61% of Trigueño Puerto Ricans report that their father knew how to read, compared to 75% of White Puerto Ricans. In Puerto Rico, White and Mestizo older adults have higher levels of education than their Black and Trigueño counterparts. Trigueño older adults have particularly low levels of education. While about a quarter of White (26%) and Mestizo (25%) individuals had more than a high school education, just 20% of Black peers and 13% of Trigueño did. Non-white Puerto Ricans are much more likely to report discrimination than White Puerto Ricans, with Black Puerto Ricans most likely (14%) of all groups to report experiences of discrimination.

### 4.2 Discrimination

Color Discrimination (Table 2) –For logistic regression models estimating the likelihood of individuals ever experiencing color discrimination, we report marginal effects (or average differences in probabilities) for ease of interpretation and to have a sense of the magnitude of effects. Black, Trigueño and Mestizo Puerto Ricans are much more likely to report experiencing color discrimination than white Puerto Ricans. Model 1 shows that the probability of experiencing color discrimination is greatest for Black Puerto Ricans – they are 12 percentage points more likely than white individuals to experience it, while Mestizo and Trigueño Puerto Ricans are 6.5 and 3.9 percentage points more likely to report color discrimination than white Puerto Ricans, respectively. These strong associations persist even after we adjust for respondent education, family status and background (model 2). Women are 2.4 percentage points less likely than men to report color discrimination. The sex-specific models reveal some interesting similarities and differences. Black Puerto Rican men are most likely to report (experience of) color discrimination: 13 percentage points more than white Puerto Rican men. Black Puerto Rican women are also most likely to report color discrimination - 11 percentage points more than white women. Trigueño Puerto Rican men and women are also more likely to report color discrimination than white Puerto Ricans (by 4.3 and 3.7 percentage points, respectively), although less so than Black Puerto Ricans. Education and family background help explain some of Black men's color discrimination experiences, but do not appear to help explain Black women's discrimination experiences. Finally and surprisingly, Mestizo men report considerable color discrimination relative to white men (13 percentage points more) and at levels comparable to Black men, but Mestizo women do not. They are as likely to report color discrimination as white women.

**Table 2:** Marginal Effects (average difference in probabilities) from Logistic Regression Models Predicting Color Discrimination experienced by Puerto Rican older adults, PREHCO (2002).

	(1)	(2)	(3)	(4)	(5)	(6)
	Pooled Model 1	Pooled Model 2	Males Model 1	Males Model 2	Females Model 1	Females Model 2
**Color/Race (reference: white)**						
Negro/a	0.11***	0.11***	0.10*	0.11*	0.10**	0.10**
	(0.029)	(0.029)	(0.044)	(0.044)	(0.034)	(0.033)
Trigueño/a or Mulato/a	0.040***	0.042***	0.045**	0.047**	0.038***	0.038***
	(0.0087)	(0.0087)	(0.016)	(0.016)	(0.0095)	(0.0093)
Mestizo	0.065**	0.062**	0.12**	0.11**	0.0092	0.0092
	(0.023)	(0.022)	(0.043)	(0.041)	(0.012)	(0.011)
**Socio-demographics**	Yes	Yes	Yes	Yes	Yes	Yes
**Education variables**		Yes		Yes		Yes
N	3575	3575	1429	1429	2146	2146

Notes: Weighted analysis. Standard errors in parentheses. Socio-demographic variables are age, age-squared, foreign-born, married and rural childhood. Education variables are own education, father literacy. Pooled models include female. + p < 0.10; *p < 0.05 ; **p < 0.01; ***p < 0.001.

### 4.3 Cognitive health - cognitive functioning and cognitive change

Cognitive scores at baseline (Table 3) – We use generalized linear models to estimate cognitive functioning at baseline. No race or color group appears to be at a disadvantage at baseline in the pooled models. Women have higher scores than men. All levels of education are clearly protective, and older adults who come from more privileged backgrounds (their fathers were literate) are at an even greater advantage. In formal tests, color/race and sex interaction effects for baseline neurocognitive functioning are statistically significant. In sex-specific models, there are few color/race differences among men, and standard errors are large. With education included in the model, Trigueño men outperform white men in terms of cognitive functioning at baseline. Their advantage is of only marginal statistical significance and its size is about one-third of the advantage that men with a secondary education have over those with a primary education or less. Men who spent their childhood in the countryside are particularly disadvantaged, even beyond their own educational attainment. Among women however, Trigueña women underperform white women in terms of cognitive testing at baseline (the size is about one-third of the advantage that the secondary-educated have over the primary- or non-educated). The coefficient decreases in models adjusting for education, but given the wide confidence interval, we cannot say anything with statistical significance about mediation effects. With a larger sample size, this could be tested with more formal mediation techniques. Women whose fathers were literate have an advantage in terms of cognitive functioning. For men and women, the coefficients of color/race do not change with the inclusion of perceived color discrimination in Models 4. Such results would be inconsistent with mediation, but the confidence intervals are wide enough that we cannot comment with statistical significance. This can be tested with larger sample sizes and more formal mediation techniques in the future.

**Table 3:** Marginal effects from generalized linear Regression Models Predicting Cognition (Mini-Mental Cabán) in Puerto Rican older adults, PREHCO (2002).

	Pooled				Males				Females			
	Model 1	Model 2	Model 3	Model 4	Model 1	Model 2	Model 3	Model 4	Model 1	Model 2	Model 3	Model 4
**Color/Race (reference: white)**												
Negro/a	−0.25	−0.26	−0.21	−0.19	−0.19	−0.23	−0.083	−0.034	−0.25	−0.24	−0.27	−0.28
	(0.27)	(0.27)	(0.25)	(0.25)	(0.50)	(0.50)	(0.45)	(0.46)	(0.27)	(0.27)	(0.25)	(0.25)
Trigueño/a or Mulato/a	−0.14	−0.13	0.0091	0.018	0.13	0.17	0.28+	0.30+	−0.32*	−0.32*	−0.16	−0.16
	(0.12)	(0.12)	(0.12)	(0.12)	(0.17)	(0.16)	(0.16)	(0.16)	(0.16)	(0.16)	(0.16)	(0.16)
Mestizo/a	0.058	0.062	0.10	0.12	0.19	0.24	0.24	0.29	−0.042	−0.050	0.061	0.060
	(0.24)	(0.23)	(0.21)	(0.21)	(0.37)	(0.35)	(0.31)	(0.30)	(0.27)	(0.27)	(0.24)	(0.24)
**Own education (reference: 6th grade or less)**												
7-12^th^ grade			0.81***	0.81***			0.86***	0.87***			0.81***	0.81***
			(0.12)	(0.12)			(0.20)	(0.20)			(0.16)	(0.16)
More than 12^th^ grade			1.36***	1.37***			1.28***	1.29***			1.47***	1.47***
			(0.15)	(0.15)			(0.23)	(0.23)			(0.17)	(0.17)
**Father literacy (reference: father not literate)**												
Father literate			0.36**	0.36**			0.25	0.26			0.44*	0.44*
			(0.13)	(0.13)			(0.19)	(0.19)			(0.18)	(0.17)
Father literacy unknown			0.25	0.43+			0.60+	0.61+			0.32	0.32
			(0.23)	(0.23)			(0.36)	(0.35)			(0.28)	(0.28)
**Perceived Color Discrimination**				−0.19				−0.37				0.080
				(0.26)				(0.39)				(0.28)
Socio-demographics	Yes	Yes	Yes	Yes	Yes	Yes	Yes	Yes	Yes	Yes	Yes	Yes
Health conditions & behaviors		Yes	Yes	Yes		Yes	Yes	Yes		Yes	Yes	Yes
N	3575	3575	3575	3575	1429	1429	1429	1429	2146	2146	2146	2146

Notes: Weighted analysis. Standard errors in parentheses. Socio-demographic variables are age, age-squared, foreign-born, married and rural childhood. Health conditions and behaviors are diabetes, high blood pressure, ever smoke, exercise. Pooled models include female. + p < 0.10; *p < 0.05 ; **p < 0.01; ***p < 0.001.

Cognitive decline between wave 1 and wave 2 (Table 4) – We use generalized linear models to estimate cognitive decline between the two waves of data collection. There appear to be some color/race differences in the pooled sample. Relative to white Puerto Ricans, Mestizo Puerto Ricans experience less cognitive decline. The effect is of marginal statistical significance. Mestizo individuals’ advantage is significant in size, about two-thirds as large as the advantage of the college-educated over individuals with primary schooling. These differences endure even once models are adjusted for education and perceived discrimination. Given the wide confidence intervals, we cannot draw conclusions of mediation effects with statistical significance. With a larger sample size, this could be tested with more formal mediation techniques. In the pooled sample, women appear more likely to show cognitive decline, but differences by sex are not detectable in adjusted models. As expected, education protects against cognitive decline although this is only true for individuals earning a post-secondary education.

**Table 4:** Marginal effects from generalized linear Regression Models Predicting Cognitive Decline in Puerto Rican older adults (Wave 1- Wave 2), PREHCO (2002, 2006).

	Pooled				Males				Females			
	Model 1	Model 2	Model 3	Model 4	Model 1	Model 2	Model 3	Model 4	Model 1	Model 2	Model 3	Model 4
**Color/Race (reference: white)**												
Negro/a	0.37	0.31	0.31	0.37	0.78+	0.72+	0.74+	0.87*	0.0056	−0.10	−0.11	−0.11
	(0.27)	(0.26)	(0.27)	(0.27)	(0.42)	(0.39)	(0.40)	(0.41)	(0.36)	(0.35)	(0.35)	(0.34)
Trigueño/a or Mulato/a	0.12	0.12	0.049	0.068	0.20	0.23	0.21	0.24	0.063	0.040	−0.032	−0.032
	(0.16)	(0.15)	(0.15)	(0.16)	(0.26)	(0.25)	(0.25)	(0.25)	(0.19)	(0.19)	(0.19)	(0.19)
Mestizo/a	−0.54+	−0.52+	−0.54+	−0.50+	−0.29	−0.13	−0.16	−0.023	−0.80*	−0.80*	−0.77*	−0.77*
	(0.31)	(0.31)	(0.31)	(0.30)	(0.49)	(0.46)	(0.46)	(0.42)	(0.34)	(0.35)	(0.34)	(0.34)
**Own education (reference: 6th grade or less)**												
7-12^th^ grade			−0.16	−0.15			−0.15	−0.14			−0.081	−0.081
			(0.18)	(0.18)			(0.32)	(0.32)			(0.21)	(0.21)
More than 12^th^ grade			−0.77***	−0.76***			−0.41	−0.38			−0.97***	−0.97***
			(0.21)	(0.21)			(0.38)	(0.38)			(0.23)	(0.23)
**Father literacy (reference: father not literate)**												
Father literate			0.19	0.21			0.29	0.34			0.13	0.13
			(0.19)	(0.19)			(0.30)	(0.30)			(0.22)	(0.22)
Father literacy unknown			0.25	0.14			0.41	0.43			−0.030	−0.029
			(0.38)	(0.38)			(0.70)	(0.70)			(0.42)	(0.42)
**Perceived Color Discrimination**				−0.48				−0.95+				0.0098
				(0.37)				(0.57)				(0.36)
Socio-demographics	Yes	Yes	Yes	Yes	Yes	Yes	Yes	Yes	Yes	Yes	Yes	Yes
Health conditions & behaviors		Yes	Yes	Yes		Yes	Yes	Yes		Yes	Yes	Yes
N	2681	2681	2681	2681	1013	1013	1013	1013	1668	1668	1668	1668

Notes: Weighted analysis. Standard errors in parentheses. Socio-demographic variables are age, age-squared, foreign-born, married and rural childhood. Health conditions and behaviors are diabetes, high blood pressure, ever smoke, exercise. Pooled models include female. + p < 0.10; *p < 0.05 ; **p < 0.01; ***p < 0.001.

Here again, the sex-specific models are illustrative. Formal color/race and sex interaction effects for cognitive decline are not statistically significant. Black Puerto Rican men experience greater - albeit marginally significant - cognitive decline between waves than white Puerto Rican men. Trigueño men also appear to be at a disadvantage, but the effects are not statistically significant. Education surprisingly did not appear to protect men against cognitive decline in our models. Among women, post-secondary education does protect women from cognitive decline. Mestiza Puerto Rican women appear to enjoy significant protection against cognitive decline relative to white Puerto Rican women, and there are no other color- or race-related associations. The size of the Mestiza women's advantage is about four-fifths as large as the advantage of college-educated women over women with primary schooling. Both the coefficients for Black men (marking disadvantage) and Mestiza women (marking relative advantage) do not change with the inclusion of education variables. While such results would be inconsistent with our mediation hypotheses for cognitive decline, the confidence intervals are sufficiently wide that we cannot comment on these with statistical significance. These can be tested with larger sample sizes and more formal mediation techniques in the future. Such sample sizes and mediation techniques would be helpful to unravel the role of perceived discrimination in color disparities of cognitive decline.

## 5 Discussion

Overall, we find evidence for color/race disparities in cognitive health among older adults in Puerto Rico. First, we find some support for the hypothesis that individuals identifying with marginalized colors/races experience poorer cognitive health outcomes (hypothesis a1). Trigueña Puerto Rican women are disadvantaged in baseline neurocognitive functioning relative to white Puerto Rican women. Marginalized groups also experience greater cognitive changes over time. Between the two waves of data, Black Puerto Rican men experience greater cognitive decline, relative to white Puerto Rican men. Mestizo women appear to be less likely to experience cognitive decline relative to white Puerto Ricans. The results for Mestizo Puerto Ricans may be related to more fluid boundaries between white and Mestizo groups and/or selectivity into Mestizo identification, as found in previous Latin America research.

Second, we explored two alternative hypotheses for color and gender: that women experience stronger color stratification than men (hypothesis b1); and that they experience comparable color stratification (hypothesis b2). We found evidence for both in formal tests of sex and race interaction effects. Women experience stronger color health gradients for baseline cognitive functioning than men. However, color- and race-based health patterns of cognitive decline for men and women are not significantly different from one another.

Third, for this population of older Puerto Rican adults, we find some support that family social background and one's own education as mediators of the impact of color/race and cognitive health (hypothesis c). Father's literacy is strongly associated with better cognitive health at baseline, but not with cognitive decline. An individual's own level of education is also strongly associated with neurocognitive functioning at baseline and cognitive decline. Controlling for education reduces by half the significant Trigueño disadvantage in female baseline cognitive performance; however, education does not appear to play a mediating role in explaining the faster cognitive *decline* of lower-status color/race groups.

Finally, while we find strong color/race disparities in color discrimination overall and men and women separately, color discrimination is not strongly associated with cognitive health and we cannot conclude whether it mediates the impact of color and either of our cognitive health measures: neurocognitive functioning at baseline, cognitive decline between waves (hypothesis d). Like previous studies of African Americans and Latinos in Latin America, color is strongly associated with reports of perceived discrimination by older Puerto Ricans in our study. Non-white Puerto Ricans' higher likelihood to report discrimination relative to white Puerto Ricans is not explained by differences in economic or social standing. However, we do not find evidence that color discrimination is itself associated with cognitive health. This likely reflects the challenges of untangling discrimination's influence on cognition. Highly-educated Black older adults are more likely to experience color discrimination, and so the two potential pathways between discrimination and cognitive outcomes – education and stress – operate in different directions. These results and the advanced age of our sample supports the contention that color stratification of Puerto Rico is deeply rooted in Puerto Rico, and separate gender spheres have not protected Puerto Ricans of color. We provide new evidence that colorism is a complex institution that impacts both Puerto Rican men and women (Gravlee, 2005; Gravlee et al., 2005; Landale & Oropesa, 2005).

Our study has limitations. First, our sample excludes proxy respondents who had low MMC scores at baseline in 2002 and are older on average. We may underestimate the color stratification of cognitive health in Puerto Rico if marginalized groups systematically scored below the PREHCO cut-off and were then excluded from our analysis of baseline cognition. Or we may overestimate inequality if the older, excluded respondents are more likely to identify with the advantaged groups (white, Mestizo Puerto Ricans) as would be the case if there are racial disparities in longevity, for example. Overall, these may be minor issues, as the cut-off does not appear to truncate significantly the MMC score distributions (see Fig. 2). Next and importantly, our cognition and color/race measures are limited. A larger battery of neurocognitive tests, application of dementia algorithms or a gold-standard physician diagnosis of dementia or cognitive impairment would be preferred (Gianattasio et al., 2020; Langa et al., 2020). Additional explicit, interviewer- and respondent-assessed skin tone and color/race classification would be helpful (Roth, 2016). Also, our study examines Puerto Ricans living in Puerto Rico and cannot account for those who migrated to the mainland United States. Given previous research of negative education selectivity of Puerto Rican migration to the U.S. (Feliciano, 2005) and the color/race disparities in educational attainment found in our study, more research is needed to understand whether and how migration influences color/race disparities in education, cognitive health and overall health among Puerto Ricans.

Nevertheless, our results of color/race stratification of cognitive health in Puerto Rico are intriguing and lead to additional questions. First, education is a primary modifiable risk factor for ADRD, yet the categorical variable we used does not appear to contribute significantly to explaining cognitive decline among older Puerto Rican men in covariate-adjusted models. This surprising finding contrasts with education's strong role in explaining disparities at baseline and requires further inquiry. Second, the greater cognitive decline experienced by Black men relative to white men is stark. In just four years between the two waves of PREHCO, Black men experienced 0.78 more points of cognitive decline than white men in Puerto Rico. The entire MMC scale is only 20 points. The inclusion of health conditions and behaviors education, father literacy and perceived color discrimination does not appear to attenuate this disparity. Similarly, Mestiza women's advantage and slower cognitive decline relative to white women also endures despite the inclusion of key covariates. Understanding why Black Puerto Rican men experience greater cognitive decline (and why Mestiza women experience less) than white Puerto Ricans is essential for understanding how we can address and remedy these disparities. More data will be important for these efforts. A new wave of PREHCO is currently in the field and will offer more opportunities to examine color inequalities in Puerto Rico.

To answer these and other questions, a careful examination of contexts and life course experiences is needed. In the U.S., race- and color stratification of health and well-being is closely linked to racial regimes of segregation, education (Hannon et al., 2013; Okonofua et al., 2016), and mass incarceration (King & Johnson, 2016; Monk, 2019). While influenced by U.S. colonialism and migrations, Puerto Rico's history and racial regimes are distinct and may have different consequences for understanding population disparities in ADRD. A sustained examination of the life course may help unearth explanations. Previous research on Puerto Rico findings that early life health conditions influence adult heart disease (McEniry & Palloni, 2010), as well as adult diabetes and hypertension (McEniry, 2011). While we have been able to look at family background through father literacy and rural childhood, we might be able to examine early life health conditions in other ways. Prior ADRD research relates how factors such as childhood stress and adversity (e.g. poor childhood nutrition (Abbott et al., 1998), household SES, death of parent (M. C. Norton et al., 2009; Whalley et al., 2013), and head injury, etc.) can contribute to higher ADRD risk, especially for those experiencing multiple adverse events (Tani et al., 2020). While we have accounted here for diabetes (Xu et al., 2009)), other sources of midlife stress and adversity (e.g. work characteristics, household SES, death of child (Umberson et al., 2019) may also contribute to ADRD risk. These factors may work through greater likelihood of depression and chronic stress (Byers & Yaffe, 2011; Leonard, 2007), decreasing cognitive reserve and raising ADRD risk.

## CRediT authorship contribution statement

**Mao-Mei Liu:** Conceptualization, Methodology, Software, Data curation, Writing – original draft. **Michael Crowe:** Conceptualization, Writing – review & editing. **Edward E. Telles:** Conceptualization, Writing – review & editing. **Ivonne Z. Jiménez-Velázquez:** Writing – review & editing. **William H. Dow:** Conceptualization, Methodology, Writing – review & editing.

## Declaration of competing interest

We have no known conflict of interests.

## Figures and Tables

**Appendix:** Table 1Sample Statistics of Puerto Rican Older Adults by Proxy status presented as Weighted Percentages (%) and Means (Standard Errors), PREHCO (2002)

	No Proxy	Proxy	
**Health behaviors**			
Hypertension (self-report) (%)	58	53	
Diabetes (self-report) (%)	28	30	
Ever smoke (%)	34	34	
Exercise (%)	43	10	***
**Education** (%)			***
6^th^ grade or less	40	69	
7^th^ to 12^th^ grade	40	23	
More than 12^th^ grade	20	8	
**Individual factors**			
Age	69.9 (0.2)	77.3 (0.8)	***
Female (%)	56	55	
Foreign-born (%)	4	3	
Married (%)	53	34	***
**Number of observations (unweighted)**	3713	578	

**Appendix:** Table 2Full table of Marginal Effects (average difference in probabilities) from Logistic Regression Models Predicting Color Discrimination experienced by Puerto Rican older adults, PREHCO (2002)

	(1)		(2)	(3)	(4)	(5)	(6)
	Pooled Model 1		Pooled Model 2	Males Model 1	Males Model 2	Females Model 1	Females Model 2
**Color/Race (reference: white)**							
Negro/a	0.11***		0.11***	0.10*	0.11*	0.10**	0.10**
	(0.029)		(0.029)	(0.044)	(0.044)	(0.034)	(0.033)
Trigueño/a or Mulato/a	0.040***		0.042***	0.045**	0.047**	0.038***	0.038***
	(0.0087)		(0.0087)	(0.016)	(0.016)	(0.0095)	(0.0093)
Mestizo	0.065**		0.062**	0.12**	0.11**	0.0092	0.0092
	(0.023)		(0.022)	(0.043)	(0.041)	(0.012)	(0.011)
***Socio-demographic variables***							
Female	−0.026***		−0.025***				
	(0.0074)		(0.0073)				
Age	0.0073		0.0067	0.030	0.027	−0.00073	−0.00090
	(0.0075)		(0.0070)	(0.019)	(0.018)	(0.0056)	(0.0052)
Age squared	−0.000056		−0.000050	−0.00022	−0.00019	0.0000017	0.0000036
	(0.000052)		(0.000049)	(0.00013)	(0.00013)	(0.000038)	(0.000035)
Foreign-born	0.0039		0.0018	−0.019	−0.022	0.015	0.013
	(0.017)		(0.017)	(0.035)	(0.034)	(0.016)	(0.015)
Rural childhood	−0.020**		−0.014+	−0.037**	−0.026+	−0.0085	−0.0071
	(0.0072)		(0.0074)	(0.014)	(0.014)	(0.0066)	(0.0068)
Married	−0.0027		−0.0036	0.0087	0.0073	−0.0089	−0.0084
	(0.0075)		(0.0074)	(0.015)	(0.016)	(0.0079)	(0.0076)
**Own education** (reference: 6th grade or less)							
7-12^th^ grade		0.0031			0.011		−0.0026
		(0.0080)			(0.015)		(0.0081)
More than 12^th^ grade		0.018			0.027		0.011
		(0.012)			(0.022)		(0.011)
**Father literacy** (reference: father not literate)							
Father literate		0.013+			0.024+		0.0018
		(0.0073)			(0.014)		(0.0080)
Father literacy unknown		−0.0016			0.027		−0.017+
		(0.011)			(0.029)		(0.0091)
N	3575	3575		1429	1429	2146	2146

Notes: Weighted analysis. Standard errors in parentheses. + p < 0.10; *p < 0.05; **p < 0.01; ***p < 0.001.

**Appendix:** Table 3Full table of Marginal effects from generalized linear Regression Models Predicting Cognition (Mini-Mental Cabán) in Puerto Rican older adults, PREHCO (2002)

	Pooled				Males				Females			
	Model 1	Model 2	Model 3	Model 4	Model 1	Model 2	Model 3	Model 4	Model 1	Model 2	Model 3	Model 4
**Color/Race (reference: white)**												
Negro/a	−0.25	−0.26	−0.21	−0.19	−0.19	−0.23	−0.083	−0.034	−0.25	−0.24	−0.27	−0.28
	(0.27)	(0.27)	(0.25)	(0.25)	(0.50)	(0.50)	(0.45)	(0.46)	(0.27)	(0.27)	(0.25)	(0.25)
Trigueño/a or Mulato/a	−0.14	−0.13	0.0091	0.018	0.13	0.17	0.28+	0.30+	−0.32*	−0.32*	−0.16	−0.16
	(0.12)	(0.12)	(0.12)	(0.12)	(0.17)	(0.16)	(0.16)	(0.16)	(0.16)	(0.16)	(0.16)	(0.16)
Mestizo/a	0.058	0.062	0.10	0.12	0.19	0.24	0.24	0.29	−0.042	−0.050	0.061	0.060
	(0.24)	(0.23)	(0.21)	(0.21)	(0.37)	(0.35)	(0.31)	(0.30)	(0.27)	(0.27)	(0.24)	(0.24)
**Own education** (reference: 6th grade or less)												
7-12^th^ grade			0.81***	0.81***			0.86***	0.87***			0.81***	0.81***
			(0.12)	(0.12)			(0.20)	(0.20)			(0.16)	(0.16)
More than 12^th^ grade			1.36***	1.37***			1.28***	1.29***			1.47***	1.47***
			(0.15)	(0.15)			(0.23)	(0.23)			(0.17)	(0.17)
**Father literacy** (reference: father not literate)												
Father literate			0.36**	0.36**			0.25	0.26			0.44*	0.44*
			(0.13)	(0.13)			(0.19)	(0.19)			(0.18)	(0.17)
Father literacy unknown			0.25	0.43+			0.60+	0.61+			0.32	0.32
			(0.23)	(0.23)			(0.36)	(0.35)			(0.28)	(0.28)
**Perceived Color Discrimination**				−0.19				−0.37				0.080
				(0.26)				(0.39)				(0.28)
***Socio-demographic variables***												
Female	0.41***	0.46***	0.50***	0.49***								
	(0.12)	(0.13)	(0.13)	(0.12)								
Age	−0.11	−0.12	−0.10	−0.10	−0.47**	−0.53**	−0.53**	−0.52**	0.11	0.12	0.14	0.14
	(0.12)	(0.12)	(0.12)	(0.12)	(0.18)	(0.17)	(0.17)	(0.17)	(0.15)	(0.15)	(0.14)	(0.14)
Age squared	0.00035	0.00044	0.00040	0.00039	0.0029*	0.0034**	0.0035**	0.0034**	−0.0013	−0.0013	−0.0014	−0.0014
	(0.00081)	(0.00080)	(0.00079)	(0.00079)	(0.0012)	(0.0012)	(0.0012)	(0.0012)	(0.00099)	(0.00099)	(0.00097)	(0.00096)
Foreign-born	0.020	0.015	−0.042	−0.042	0.23	0.19	0.013	0.0026	−0.081	−0.073	−0.058	−0.059
	(0.28)	(0.28)	(0.28)	(0.28)	(0.39)	(0.37)	(0.38)	(0.37)	(0.39)	(0.39)	(0.39)	(0.39)
Rural childhood	−0.59***	−0.58***	−0.17	−0.17	−0.78***	−0.81***	−0.44**	−0.45**	−0.44**	−0.41**	0.033	0.034
	(0.11)	(0.11)	(0.11)	(0.11)	(0.17)	(0.16)	(0.16)	(0.16)	(0.14)	(0.14)	(0.13)	(0.13)
Married	0.30*	0.30*	0.26*	0.26*	0.25	0.24	0.18	0.19	0.31+	0.30+	0.28+	0.28+
	(0.12)	(0.12)	(0.12)	(0.12)	(0.18)	(0.17)	(0.17)	(0.17)	(0.17)	(0.17)	(0.16)	(0.16)
***Health conditions & behaviors***												
Diabetes		0.13	0.13	0.13		0.38*	0.35*	0.35*		−0.059	−0.016	−0.017
		(0.13)	(0.13)	(0.13)		(0.17)	(0.17)	(0.17)		(0.18)	(0.18)	(0.18)
Hypertension		−0.095	−0.14	−0.14		−0.16	−0.23	−0.23		−0.044	−0.052	−0.052
		(0.12)	(0.11)	(0.11)		(0.16)	(0.16)	(0.16)		(0.16)	(0.16)	(0.16)
Ever smoke		−0.039	−0.0017	0.0010		0.084	0.085	0.091		−0.20	−0.095	−0.095
		(0.13)	(0.12)	(0.12)		(0.16)	(0.15)	(0.15)		(0.20)	(0.18)	(0.18)
Exercise		0.30**	0.085	0.084		0.49**	0.33*	0.33*		0.19	−0.085	−0.084
		(0.11)	(0.11)	(0.11)		(0.17)	(0.16)	(0.16)		(0.14)	(0.14)	(0.14)
N	3575	3575	3575	3575	1429	1429	1429	1429	2146	2146	2146	2146

Notes: Weighted analysis. Standard errors in parentheses. + p < 0.10; *p < 0.05 ; **p < 0.01; ***p < 0.001.

**Appendix:** Table 4Full table of Marginal effects from generalized linear Regression Models Predicting Cognitive Decline in Puerto Rican older adults (Wave 1- Wave 2), PREHCO (2002, 2006).

	Pooled				Males							Females							
	Model 1	Model 2	Model 3	Model 4	Model 1	Model 2		Model 3		Model 4		Model 1		Model 2		Model 3		Model 4	
**Color/Race (reference: white)**																			
Negro/a	0.37	0.31	0.31	0.37	0.78+	0.72+		0.74+		0.87*		0.0056		−0.10		−0.11		−0.11	
	(0.27)	(0.26)	(0.27)	(0.27)	(0.42)	(0.39)		(0.40)		(0.41)		(0.36)		(0.35)		(0.35)		(0.34)	
Trigueño/a or Mulato/a	0.12	0.12	0.049	0.068	0.20	0.23		0.21		0.24		0.063		0.040		−0.032		−0.032	
	(0.16)	(0.15)	(0.15)	(0.16)	(0.26)	(0.25)		(0.25)		(0.25)		(0.19)		(0.19)		(0.19)		(0.19)	
Mestizo/a	−0.54+	−0.52+	−0.54+	−0.50+	−0.29	−0.13		−0.16		−0.023		−0.80*		−0.80*		−0.77*		−0.77*	
	(0.31)	(0.31)	(0.31)	(0.30)	(0.49)	(0.46)		(0.46)		(0.42)		(0.34)		(0.35)		(0.34)		(0.34)	
**Own education** (reference: 6th grade or less)																			
7-12^th^ grade			−0.16	−0.15				−0.15		−0.14						−0.081		−0.081	
			(0.18)	(0.18)				(0.32)		(0.32)						(0.21)		(0.21)	
More than 12^th^ grade			−0.77***	−0.76***				−0.41		−0.38						−0.97***		−0.97***	
			(0.21)	(0.21)				(0.38)		(0.38)						(0.23)		(0.23)	
**Father literacy** (reference: father not literate)																			
Father literate			0.19	0.21				0.29		0.34						0.13		0.13	
			(0.19)	(0.19)				(0.30)		(0.30)						(0.22)		(0.22)	
Father literacy unknown			0.25	0.14				0.41		0.43						−0.030		−0.029	
			(0.38)	(0.38)				(0.70)		(0.70)						(0.42)		(0.42)	
**Perceived Color Discrimination**				−0.48						−0.95+								0.0098	
				(0.37)						(0.57)								(0.36)	
***Socio-demographic variables***																			
Female	0.31*	0.21	0.21	0.20															
	(0.16)	(0.17)	(0.17)	(0.17)															
Age	−0.45*	−0.45*	−0.41*	−0.41*	−0.83*		−0.86**		−0.81*		−0.78*		−0.26		−0.27		−0.25		−0.25
	(0.18)	(0.17)	(0.17)	(0.18)	(0.33)		(0.33)		(0.32)		(0.33)		(0.20)		(0.19)		(0.19)		(0.19)
Age squared	0.0037**	0.0036**	0.0034**	0.0034**	0.0063**		0.0065**		0.0062**		0.0060**		0.0023+		0.0024+		0.0022+		0.0022+
	(0.0012)	(0.0012)	(0.0012)	(0.0012)	(0.0023)		(0.0023)		(0.0023)		(0.0023)		(0.0014)		(0.0013)		(0.0013)		(0.0013)
Foreign-born	−0.42	−0.38	−0.34	−0.33	−0.71		−0.76		−0.76		−0.78		−0.19		−0.20		−0.14		−0.15
	(0.34)	(0.34)	(0.33)	(0.33)	(0.63)		(0.62)		(0.61)		(0.60)		(0.39)		(0.38)		(0.37)		(0.37)
Rural childhood	0.14	0.11	−0.029	−0.039	−0.11	−0.12		−0.17		−0.19		0.34+		0.27		0.10		0.10	
	(0.15)	(0.15)	(0.16)	(0.16)	(0.25)	(0.24)		(0.26)		(0.26)		(0.18)		(0.17)		(0.19)		(0.19)	
Married	−0.083	−0.088	−0.076	−0.079	−0.025	−0.030		−0.015		−0.0091		−0.13		−0.15		−0.13		−0.13	
	(0.16)	(0.16)	(0.16)	(0.16)	(0.27)	(0.26)		(0.27)		(0.27)		(0.18)		(0.18)		(0.18)		(0.18)	
***Health conditions & behaviors***																			
Diabetes		0.37*	0.38*	0.39*		0.74**		0.75**		0.77**				0.13		0.14		0.14	
		(0.17)	(0.17)	(0.16)		(0.27)		(0.27)		(0.26)				(0.21)		(0.20)		(0.20)	
Hypertension		0.0072	0.021	0.024		−0.47*		−0.46+		−0.46+				0.36+		0.38*		0.38*	
		(0.15)	(0.15)	(0.15)		(0.24)		(0.24)		(0.24)				(0.19)		(0.19)		(0.19)	
Ever smoke		−0.064	−0.077	−0.067		−0.17		−0.18		−0.16				0.084		0.057		0.057	
		(0.17)	(0.16)	(0.16)		(0.23)		(0.23)		(0.22)				(0.22)		(0.21)		(0.21)	
Exercise		−0.33*	−0.25+	−0.25+		−0.021		0.0070		−0.0031				−0.55**		−0.44*		−0.44*	
		(0.15)	(0.15)	(0.15)		(0.24)		(0.23)		(0.23)				(0.18)		(0.18)		(0.18)	
N	2681	2681	2681	2681	1013	1013		1013		1013		1668		1668		1668		1668	

Notes: Weighted analysis. Standard errors in parentheses. + p < 0.10; *p < 0.05 ; **p < 0.01; ***p < 0.001.
